# Comparative pathogenicity of three A(H5N1) clade 2.3.4.4b HPAI viruses in blue-winged teal and transmission to domestic poultry

**DOI:** 10.1128/msphere.00021-25

**Published:** 2025-05-22

**Authors:** Tamiru N. Alkie, Carissa Embury-Hyatt, Anthony V. Signore, Frank Baldwin, Tamiko Hisanaga, Wanhong Xu, Estella Moffat, Jolene A. Giacinti, Catherine Soos, Yohannes Berhane

**Affiliations:** 1 Canadian Food Inspection Agency, National Centre for Foreign Animal Disease 558430 , Winnipeg, Manitoba, Canada; 2 Prairie Region Wildlife and Habitat Assessment Section, Canadian Wildlife Service, Environment and Climate Change Canada 731202, Delta, British Columbia, Canada; 3 Ecotoxicology and Wildlife Health Division, Environment and Climate Change Canada, Government of Canada 142123, Ottawa, Ontario, Canada; 4 Ecotoxicology & Wildlife Health Division, Science and Technology Branch, Ecotoxicology & Wildlife Health Division, Government of Canada 142123, Ottawa, Ontario, Canada; 5 Department of Veterinary Pathology, Western College of Veterinary Medicine, University of Saskatchewan 70399, Saskatoon, Saskatchewan, Canada; 6 Department of Animal Science, University of Manitoba 8664, Winnipeg, Manitoba, Canada; 7 Department of Pathobiology, University of Guelph 448200, Guelph, Ontario, Canada; Johns Hopkins University Bloomberg School of Public Health, Baltimore, Maryland, USA

**Keywords:** clade 2.3.4.4b, ducks, A(H5N1), pathogenicity, poultry, transmission

## Abstract

Long-distance migratory ducks play a critical role in the maintenance and dissemination of A(H5N1) viruses. Comparative pathogenicity studies were conducted on blue-winged teal (BWTE; *Anas discors*) using three distinct genotypes of A(H5N1) clade 2.3.4.4b viruses (A1, B1.3, and B4.1) isolated from wild ducks in Canada. Twenty-four hours post-intranasal infection of BWTE, contact turkeys and chickens were introduced into each of the groups to evaluate viral transmission. The levels of viral shedding in BWTE increased from 3 to 7 days post-infection (dpi) and continued at lower levels until 14 dpi. The A1 genotype virus (MALL/NS/22) was found to be the least pathogenic to BWTE compared to the reassortant genotypes, B4.1 (RBME/BC/22) and B1.3 (BWTE/MB/22). The B1.3 genotype was the most virulent to BWTE and caused 66.7% mortality compared to 12.5% mortality caused by the B4.1 genotype. The extent of transmission from infected BWTE to contact turkeys and chickens showed variations. Turkeys housed with BWTE infected with either virus died within 6 to 10 days post-contact (dpc). Conversely, the transmission and mortality among contact chickens varied. The highest mortality (3 out of 5) occurred in chickens exposed to BWTE infected with the B1.3 genotype. Whilst in the B4.1 genotype, 2 out of 6 chickens died, none of the chickens in the A1 genotype succumbed to infection. No shedding or seroconversion was noted in all surviving chickens. This research underscores variations in the pathogenic traits and transmissibility among the different genotypes of A(H5N1) clade 2.3.4.4b viruses. This finding is vital for understanding the role of migratory birds in the epidemiology of A(H5N1) and the need for continuous monitoring of these viruses.

**IMPORTANCE:**

The recurrent incursions of A(H5N1) clade 2.3.4.4b viruses into North America have resulted in the emergence of reassortant virus genotypes. These genotypes exhibit variations in pathogenicity and host ranges. Blue-winged teal (BWTE) are the most common dabbling ducks in North America and play a crucial role in maintaining and dispersing influenza A viruses (IAVs). In some areas, the migratory pathways of BWTE overlap with densely populated commercial poultry facilities. Despite this, the role of BWTE in the maintenance and spread of A(H5N1) is not well understood, and there is limited data on their susceptibility to A(H5N1) clade 2.3.4.4b viruses. Our study demonstrates differences in BWTE susceptibility to distinct genotypes of A(H5N1) clade 2.3.4.4b viruses. The virus transmission from infected BWTE and lethality in turkeys and chickens were also influenced by the virus genotypes. The findings suggest that BWTE could contribute to the maintenance and spread of highly pathogenic avian influenza (HPAI) viruses, and active surveillance in BWTE is essential.

## INTRODUCTION

The recurring incursions of A(H5N1) clade 2.3.4.4b highly pathogenic avian influenza (HPAI) virus from Europe into the Atlantic provinces of Canada in 2021 resulted in the widespread dispersion of the virus throughout Canada, United States, and South America, reaching the Antarctic regions by 2023 (1–5). This clade has established a panzootic state (3, 6–9), presenting an ongoing threat to avian populations that surpasses any historical data recorded for earlier clades, including clades 2.2 and 2.3.2.1 (10, 11). The extensive infections among avian hosts inhabiting diverse ecological niches have led to significant spillover events affecting numerous terrestrial and marine mammals, including dairy cattle in the United States (9, 12–15).

Notably, A(H5N1) clade 2.3.4.4b viruses have demonstrated a sustained abundance and presence across seasons in wild birds, facilitating reassortment with various low pathogenicity avian influenza (LPAI) viruses native to North America (3, 9, 16). This genetic reassortment has resulted in the emergence of novel and diverse genotypes of A(H5N1) viruses, with some of them becoming the dominant circulating viruses (3, 9). Importantly, some genotypes display increased pathogenicity and transmissibility, resulting in substantial mortality rates among seabirds that exhibit colonial breeding behavior (8, 17, 18), as well as in scavenger birds (12–21) and unprecedented die-offs in sea lions (14). Furthermore, A(H5N1) clade 2.3.4.4b viruses have frequently been isolated from apparently healthy waterfowl within the Anseriformes order, despite occasional instances of morbidity and mortality among naturally infected wild ducks during the peak of HPAI outbreak seasons (9, 22). Mallard ducks, being the most prevalent Anseriformes, are pivotal in the maintenance and long-distance dispersal of this virus (9). Recent studies indicate that there are no significant changes in the migration behaviors of wintering wild mallards infected with A(H5N1) clade 2.3.4.4b virus compared to their non-infected counterparts (23). This suggests that dabbling ducks, in general, are capable of maintaining and silently disseminating A(H5Nx) HPAI viruses (10).

A(H5Nx) representing clades 2.2, 2.3.2.1, and 2.3.4.4 has been extensively evaluated for its pathogenicity in both domestic and wild ducks, mainly in juvenile birds (10, 11, 24–28). These studies indicate that juvenile mallards exhibit more rapid disease progression and more severe outcomes compared to adult mallards (26, 27). Conversely, studies in Pekin and Muscovy ducks have demonstrated that these species showed significant susceptibility to A(H5Nx) viruses, with resultant severe pathologies and mortality rates (29, 30). Transmission studies from infected ducks to contact domestic poultry resulted in variable outcomes. The main transmission pathways of A(H5Nx) viruses to domestic poultry include rampant lateral flock-to-flock spread once introduced (31) and recurring independent introductions from wild birds (32). Recent experimental studies have shown that a mallard-origin A(H5N1) clade 2.3.4.4b virus exhibits inefficient transmission through direct contact exposure to naïve contact poultry, despite infected mallards shedding substantial quantities of the virus (33). In contrast, effective transmission to contact chickens has been observed from infected Pekin and Muscovy ducks (29, 30). This variation suggests that the highly duck-adapted nature of the isolate from mallards may influence transmission traits (27, 33, 34).

Blue-winged teal (*Anas discors*; BWTE), which inhabit the North American flyways—particularly the wetlands of prairie Canada—migrate during the winter months to Central and South America and are implicated in the dispersion and evolution of both HPAI and LPAI viruses (9, 35, 36). However, there remains a significant gap in our understanding of their susceptibility to A(H5N1) clade 2.3.4.4b viruses and their potential role in the spillover of these viruses to domestic poultry, when compared to other dabbling duck species. Notably, BWTE migration routes overlap with areas that are densely populated by commercial poultry facilities in several states across North America during the peak of the migration seasons (36). This study was designed to assess the pathogenicity of three A(H5N1) clade 2.3.4.4b viruses in BWTE, as well as their transmission potential to naïve contact turkeys and chickens. Our experimental design involved a three-species cohabitation study that reflects a multi-species exhibition farm in Canada, where the initial incursion of A(H5N1) clade 2.3.4.4b virus was reported.

## RESULTS

### Virus characterization and phylogenetics

We isolated, sequenced, and characterized three A(H5N1) clade 2.3.4.4b HPAI viruses; one of wholly Eurasian origin and two reassortant viruses. MALL/NS/22 (A1 genotype), from a mallard duck, had all gene segments originating from a Eurasian (EA) A(H5N1) HPAI virus as described (37, 38). RBME/BC/22 (B4.1 genotype), from a red-breasted merganser (RBME), contained PB2 and NP gene segments from North American (NAm) lineage, wild-bird origin LPAI viruses, and the other six segments from EA A(H5N1) HPAI viruses. BWTE/MB/22 (B1.3 genotype), from a blue-winged teal, contained four segments (PB2, PB1, PA, and NP) from NAm lineage wild-bird origin LPAI viruses, and the other four gene segments (HA, NA, M, and NS) from EA A(H5N1) HPAI viruses.

**Fig 1:**
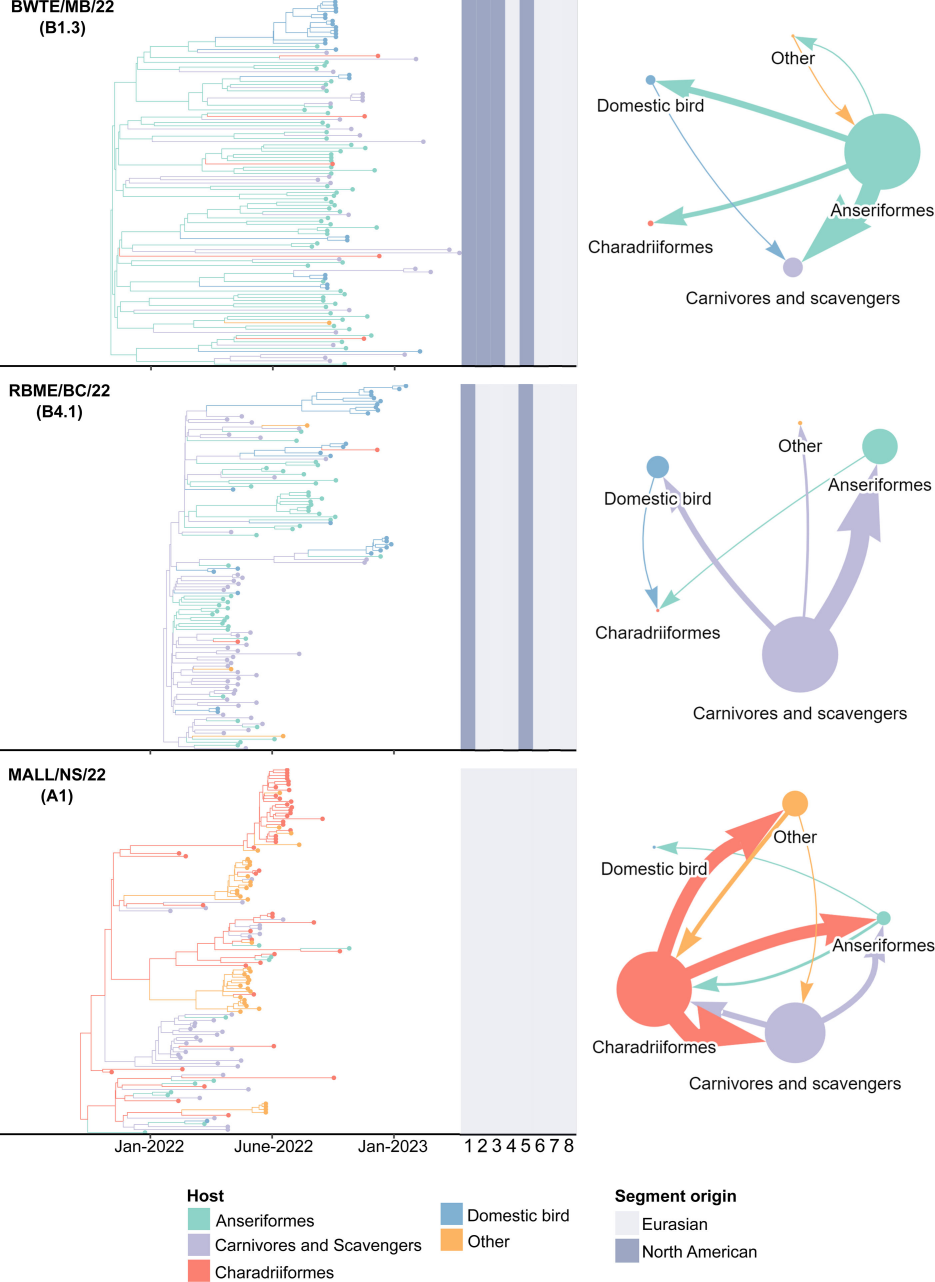
Phylogenetic trees and host dynamics of A(H5N1) viruses including BWTE/MB/2022 (B1.3 genotype), RBME/BC/2022 (B4.1 genotype), and MALL/NS/2022 (A1 genotype), and Bayesian maximum clade credibility trees based on whole-genome sequence alignments for each viral genotype examined in this study. Tiles next to each tree represent the geographic origin (either Eurasian or North American) of each genome segment (segment 1—PB2; segment 2—PB1; segment 3—PA; segment 4—HA; segment 5—NP; segment 6—NA; segment 7—M; segment 8—NS). Networks represent the host dynamics inferred from each phylogenetic tree. Arrow width summarizes jump counts between host groups (Markov jumps with >0.70 posterior probability and a Bayes factor >3.0), where each arrow is colored according to the source host. The size of each node represents the relative phylogenetic time spent in each host state (Markov rewards).

Phylogenetic trees derived from whole-genome sequences of each viral genotype (BWTE/MB/22, RBME/BC/22, and MALL/NS/22) revealed that these viruses persisted in and were transmitted by different hosts (Fig. 1). MALL/NS/22 spent time within (Markov rewards) and was transmitted by (Markov jumps) the greatest diversity of hosts, but primarily persisted in and was transmitted by Charadriiformes. In contrast, RBME/BC/22 primarily persisted in Anseriformes, carnivores, and scavengers (mesocarnivorous mammals, raptors, and corvids), but was largely transmitted by the latter. BWTE/MB/22 was almost exclusively transmitted by Anseriformes to likely scavengers. All viruses have evidence of spillover to domestic birds, while evidence of spillback from domestic birds to wild birds occurred in RBME/BC/22 and BWTE/MB/22.

### Pathogenicity of A(H5N1) in BWTE and transmission to contact turkeys and chickens

**Fig 2:**
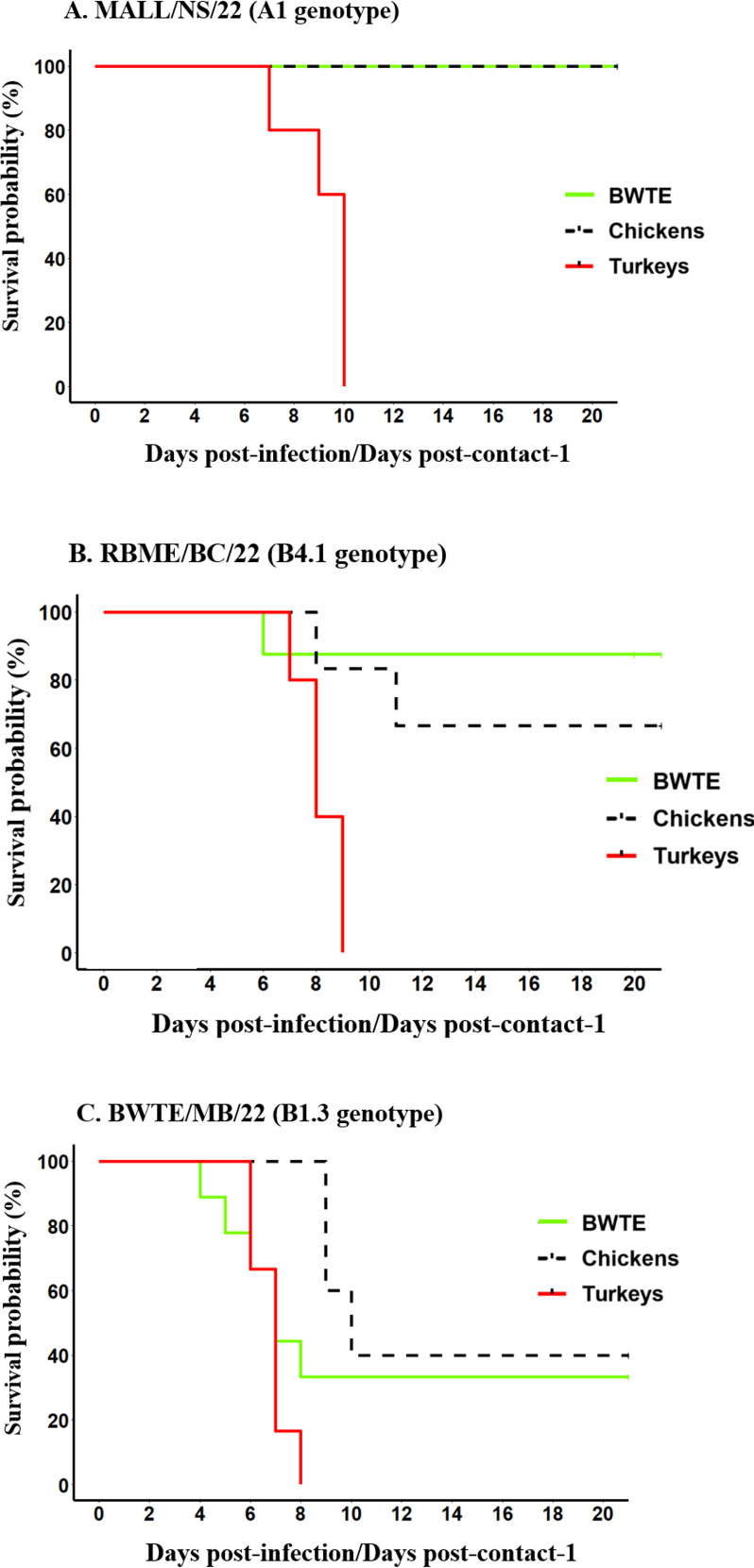
Probability of survival of ducks and contact poultry challenged with A(H5N1) clade 2.3.4.4b HPAI viruses (A, B, and C). Survival curves were generated and compared using a log-rank (Mantel-Cox) test at *P* < 0.05. BWTE was challenged with 1 × 10^6^ TCID_50_ of A(H5N1) HPAI viruses isolated in 2022 by the intranasal route. All infected birds and contact poultry were daily observed for clinical signs and mortality.

This study showed greater variation in the pathogenicity and transmission dynamics of A(H5N1) from BWTE to contact poultry. BWTE infected with MALL/NS/22 (A1 genotype) remained asymptomatic after infection, and all of them survived. Contact transmission to turkeys was efficient, with 100% mortality. Transmission and mortality were not observed in the contact chickens (Fig. 2A). RBME/BC/22 (B4.1 genotype) caused 12.5% mortality in BWTE, with diseased BWTE showing severe neurological signs, manifested by tremor, head shaking, and torticollis. All contact turkeys and 33.3% (2 out of 6) contact chickens in this group died within 9 days post-contact (dpc) (Fig. 2B). BWTE/MB/22 (B1.3 genotype) caused greater mortality in infected BWTE (66.7% mortality) and contact chickens (60% mortality, 40% survival). All turkeys either reached humane endpoints requiring euthanasia or were found dead within 8 dpc (Fig. 2C). BWTE infected with BWTE/MB/22 began dying or required euthanasia as early as 4 days post-infection (dpi), and most infected birds exhibited neurological clinical signs, indicating the neuroinvasive nature of this virus.

### Virus shedding from blue-winged teal

**Fig 3:**
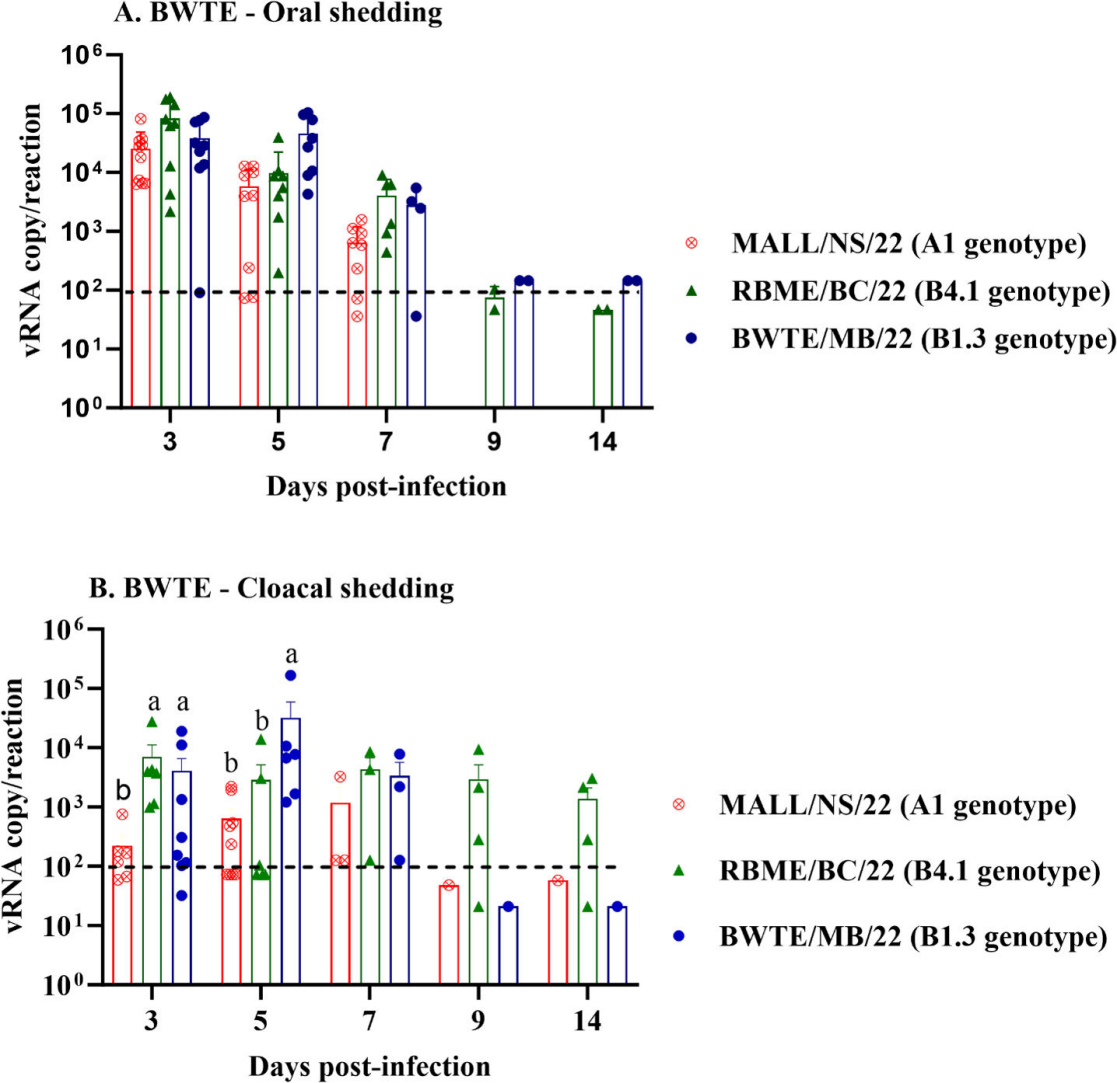
Detection, quantification, and patterns of viral RNA (vRNA) shedding in oral (A) and cloacal (B) swabs collected from BWTE. Influenza A viruses free-BWTE were challenged with 1 × 10^6^ TCID_50_ of A(H5N1) clade 2.3.4.4b HPAI viruses by the intranasal route. Oropharyngeal as well as cloacal swabs were collected from BWTE at different time points post-infection. The amount of virus load in the oropharyngeal and cloacal swabs from infected and contact birds was quantified by quantitative reverse transcription PCR (RT-qPCR) based on the linear regression of the standard curve generated from *in vitro*-synthesized RNA targeting the influenza A virus (AIV) matrix gene. Data were log transformed for evaluating normal distribution and analyzed with non-parametric analysis of variance (ANOVA), and differences were considered significant at *P* < 0.05. The results were expressed as log_10_ viral RNA copy numbers per reaction.

Differences in the magnitude and duration of viral shedding from oropharyngeal (OP) and cloacal (CL) routes were observed among the three A(H5N1) viruses (Fig. 3). BWTE infected with the reassortant viruses shed through the OP route starting at 3 dpi and for up to 2 weeks, but with a significant reduction in virus titers. No significant difference in virus shedding was detected in OP swabs among all three groups at 3 dpi. Peak virus shedding through the OP route for the MALL/NS/22 (A1 genotype) was observed at 3 dpi, and then shedding ceased at the furthest time points. At 5 and 7 dpi, BWTE infected with BWTE/MB/22 (B1.3 genotype) shed significantly higher virus load in OP swabs compared with MALL/NS/22, yet RBME/BC/22 (B4.1 genotype) infected group shed higher virus load (Fig. 3A).

MALL/NS/22-infected BWTE exhibited lower cloacal virus shedding than the other two groups from 3 to 7 dpi. The reassortant viruses replicated more efficiently in the gastrointestinal tract (GIT) and shed higher viral titers into the environment via cloacal routes. BWTE/MB/22 showed the highest cloacal shedding at 5 dpi, peaking earlier than the other viruses. Despite sharing the same 2.3.4.4b hemagglutinin protein, RBME/BC/22 demonstrated longer and more efficient GIT replication, resulting in enhanced cloacal shedding compared to both MALL/NS/22 and BWTE/MB/22 (Fig. 3B). While oral viral titers initially exceeded cloacal shedding levels across all groups during the early stages of infection, the RBME/BC/22 group exhibited a distinct pattern. In this group, cloacal shedding surpassed oral shedding at later time points, specifically between 9 and 14 dpi.

### Virus shedding from contact turkeys and chickens

**Fig 4:**
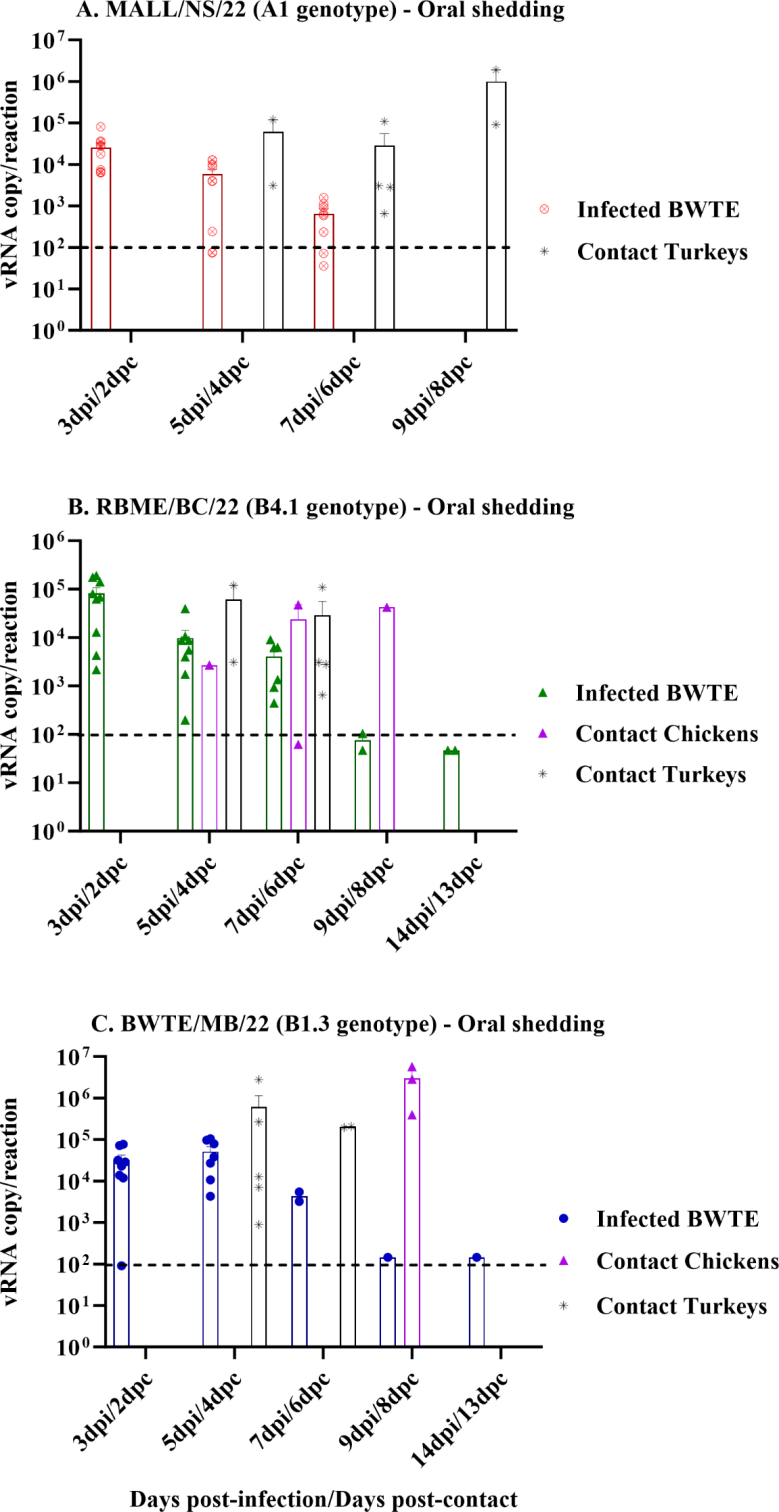
Detection and quantification of vRNA in oropharyngeal swabs (A, B, and C) collected from BWTE and contact poultry. Influenza A viruses free-BWTE were challenged with 1 × 10^6^ TCID_50_ of A(H5N1) clade 2.3.4.4b HPAI viruses by the intranasal route, and at 24 hours post-infection (hpi), contact chickens and turkeys were introduced and co-housed together with directly infected BWTE. Oral as well as cloacal swabs were collected from BWTE and poultry at different time points post-infection. The amount of virus load in the oropharyngeal swabs from infected and contact birds was quantified by RT-qPCR based on the linear regression of the standard curve generated from *in vitro*-synthesized RNA targeting the AIV matrix gene. Data were log transformed for evaluating normal distribution and analyzed with non-parametric ANOVA, and differences were considered significant at *P* < 0.05. The results were expressed as log_10_ viral RNA copy numbers per reaction.

To mimic spillover scenarios, we evaluated virus shedding in OP and CL swabs from contact chickens and turkeys. In the MALL/NS/22 (A1 genotype), no shedding was detected in chickens at all time points, but higher virus shedding was detected in the OP swabs of contact turkeys at 4, 6, and 8 dpc (Fig. 4A). In RBME/BC/22 (B4.1 genotype), OP virus shedding was higher in turkeys at 4 and 6 dpc, yet one surviving chicken continuously shed the virus until 8 dpc, before eventually dying (Fig. 4B). In the BWTE/MB/22 (B1.3 genotype), at 4 dpc, all turkeys shed higher amounts of virus in the OP swabs compared to CL swabs. At 8 dpc, 3 out of 5 chickens shed virus through the OP route (Fig. 4C), and all of these birds died or were euthanized.

**Fig 5:**
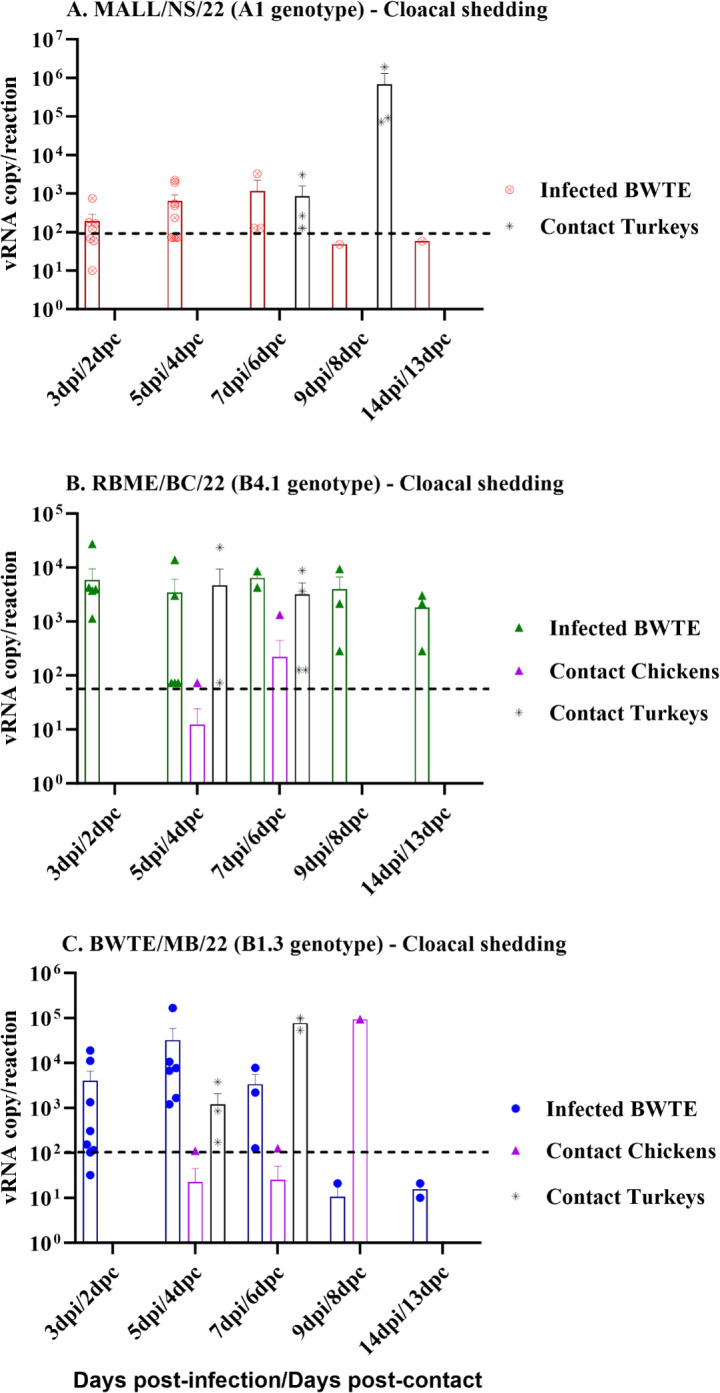
Detection and quantification of vRNA in cloacal swabs (A, B, and C) collected from BWTE and contact poultry. Influenza A viruses free-BWTE were challenged with 1 × 10^6^ TCID_50_ of A(H5N1) clade 2.3.4.4b HPAI viruses by the intranasal route, and at 24 hpi, contact chickens and turkeys were introduced and co-housed together with directly infected BWTE. Oral as well as cloacal swabs were collected from BWTE and poultry at different time points post-infection. The amount of virus load in the cloacal swabs from infected and contact birds was quantified by RT-qPCR based on the linear regression of the standard curve generated from *in vitro*-synthesized RNA targeting the AIV matrix gene. Data were log transformed for evaluating normal distribution and analyzed with non-parametric ANOVA, and differences were considered significant at *P* < 0.05. The results were expressed as log_10_ viral RNA copy numbers per reaction.

In the MALL/NS/22 group, none of the contact chickens shed virus via the CL route; however, turkeys shed large amounts of virus between 6 and 8 dpc (Fig. 5A). Remarkably, all turkeys died or were euthanized within a few days after the onset of viral shedding. In the RBME/BC/22 group (Fig. 5B), virus was detected in a CL swab of one of 5 chickens at 6 dpc. In the BWTE/MB/22 group, at 4 and 6 dpc, all turkeys shed large quantities of virus through the CL route. Among the three chickens that shed virus in the OP route, one of the chickens in this group shed higher amounts of viruses in CL swabs, only at 8 dpc (Fig. 5C).

### Pathological alterations

**Fig 6:**
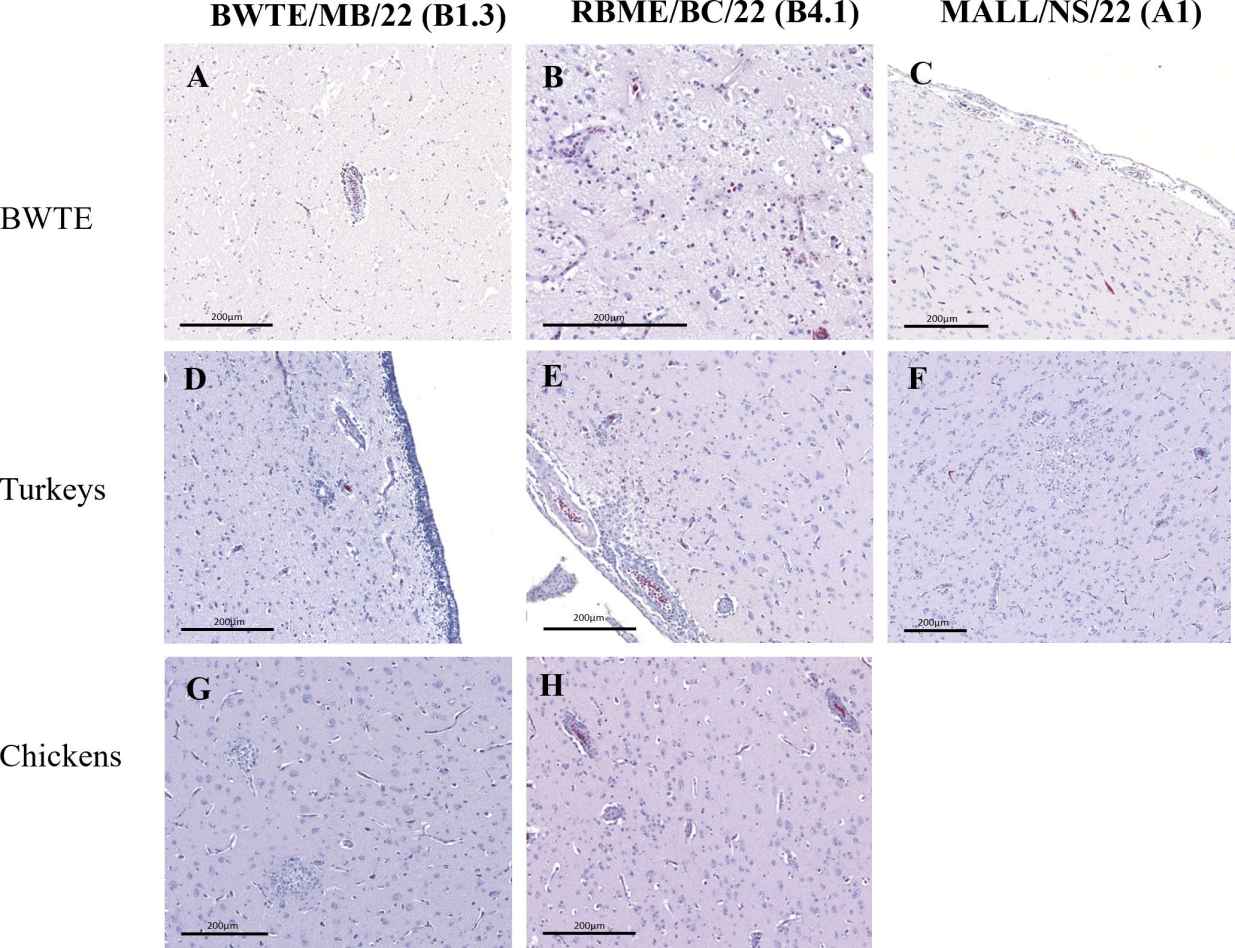
Hematoxylin and Eosin (H&E) stained brain sections from BWTE (**A through C**), contact turkeys (**D through F**), and contact specific pathogen free (SPF) chickens (**G, H**) infected with A(H5N1) clade 2.3.4.4b HPAI viruses isolated in 2022. BWTE were challenged with 1 × 10^6^ TCID_50_ of A(H5N1) HPAI viruses by the intranasal route. Twenty-four hours post-infection, turkeys and SPF chickens were introduced as contacts and allowed to cohabitate with infected ducks. Tissues collected from euthanized or acutely dead birds at 5 days post-infection/contact (dpi/dpc) were sectioned at 5 µm and stained as described in detail in Materials and Methods for microscopic evaluation. No contact chickens died in the MALL/NS/22 virus, and images were unavailable.

**Fig 7:**
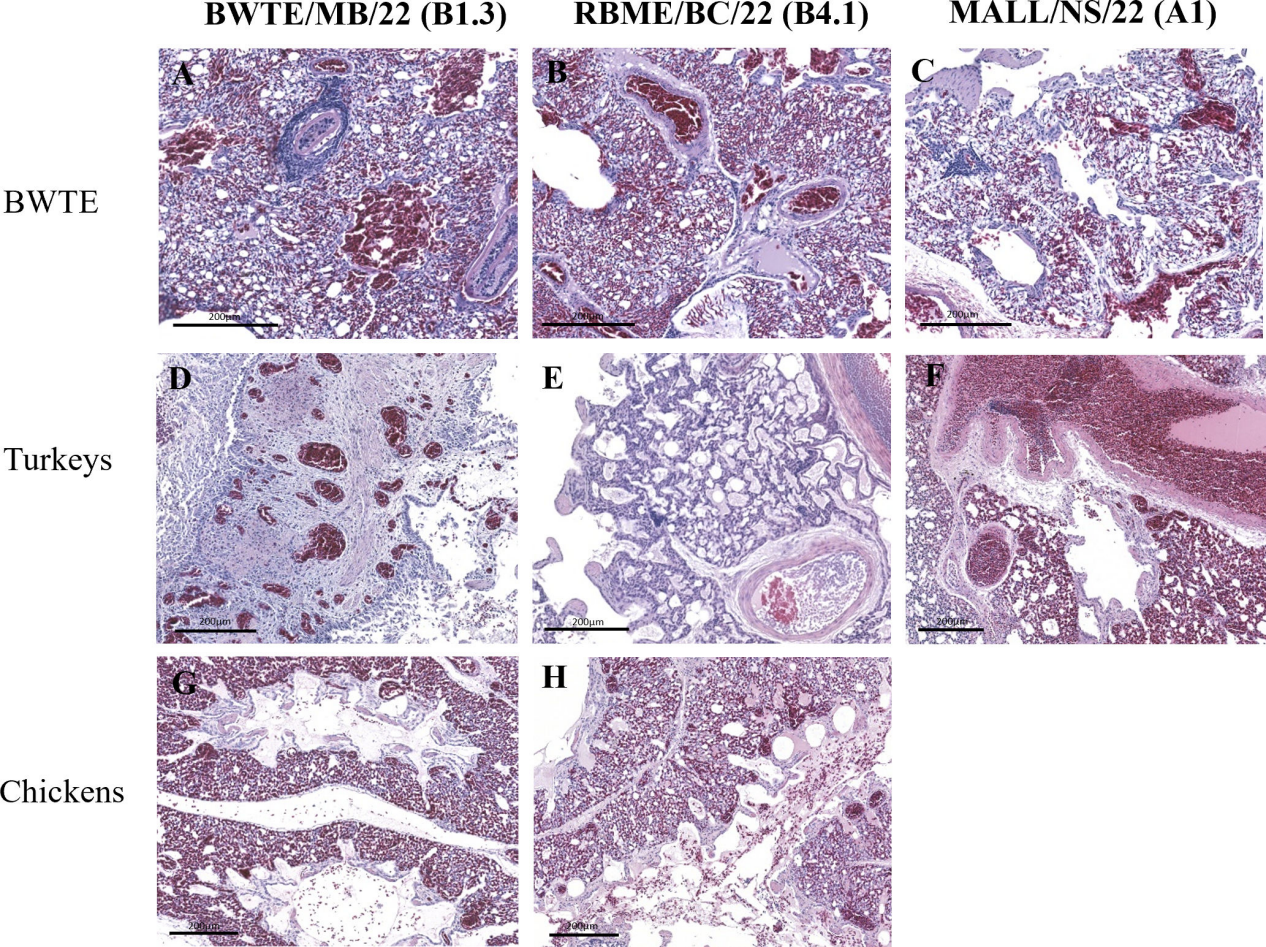
H&E stained lung sections from BWTE (**A through C**), contact turkeys (**D through F**), and contact SPF chickens (**G, H**) infected with A(H5N1) clade 2.3.4.4b HPAI viruses isolated in 2022. BWTE were challenged with 1 × 10^6^ TCID_50_ of A(H5N1) HPAI viruses by the intranasal route. Twenty-four hours post-infection, turkeys and SPF chickens were introduced as contacts and allowed to cohabitate with infected ducks. Lung specimens collected from euthanized or acutely dead birds at 5 dpi/dpc were sectioned at 5 µm and stained as described in detail in Materials and Methods for microscopic evaluation. No contact chickens died in the MALL/NS/22 virus, and images were unavailable.

As early as 3 dpi, both histopathology and immunohistochemistry (IHC) showed systemic infections in BWTE. However, both lesion severity (moderate to severe multi-organ pathologies) and influenza antigen presence were most prominent starting at 5 dpi in BWTE infected with BWTE/MB/22 (B1.3 genotype) (Table S1). Inflammatory and necrotizing lesions with lymphohistiocytic perivascular cuffs, gliosis, and neuronal degeneration were detected in the brain tissues of BWTE (Fig. 6A), turkeys (Fig. 6D), and chickens (Fig. 6G) infected with BWTE/MB/22 (B1.3 genotype). RBME/BC/22 (B4.1 genotype) infected BWTE (Fig. 6B), contact turkeys (Fig. 6E), and chickens (Fig. 6H) exhibited brain lesions like those seen in BWTE/MB/22 infection. While no apparent brain lesions were detected in MALL/NS/22 (A1 genotype) infected BWTE (Fig. 6C), multi-focal necrotizing lesions, meningitis, and perivascular cuffing by mononuclear cells were evident in the contact turkey’s brain (Fig. 6F). Histology for contact chickens in the MALL/NS/22 group was not reported as none of them died. Interstitial pneumonia and vasculitis were observed in BWTE/MB/22-infected BWTE (Fig. 7A). Lung lesions (congestion and edema) were apparent in BWTE infected with RBME/BC/22 (Fig. 7B). Occasional bronchiolitis and perivascular cuffing with mononuclear inflammatory cells were seen in BWTE infected with MALL/NS/22 (Fig. 7C). The degree and type of lung lesions described in BWTE were also visualized in turkeys (Fig. 7D through F), although one turkey infected with RBME/BC/22 had more severe lesions, including interstitial pneumonia and necrosis (Fig. 7E). Generally, lung pathologies were minimal or absent in chickens (Fig. 7G and H).

**Fig 8:**
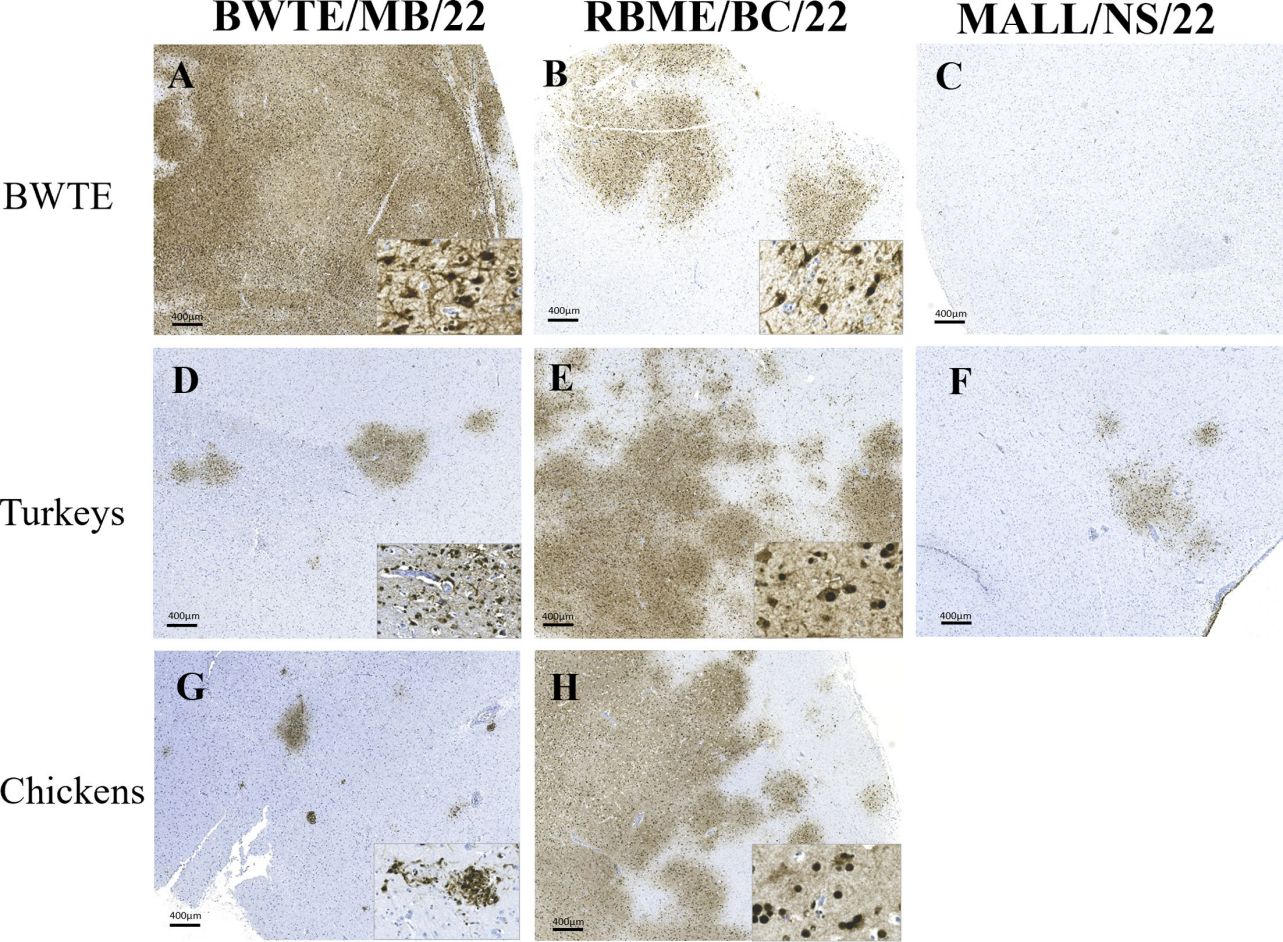
Detection of influenza A virus antigen in brain tissues of infected BWTE (**A through C**), contact turkeys (**D through F**), and contact chickens (**G, H**) with A(H5N1) clade 2.3.4.4b HPAI viruses isolated in 2022. BWTE were challenged with 1 × 10^6^ TCID_50_ of A(H5N1) HPAI viruses by the intranasal route. Twenty-four hours post-infection, turkeys and SPF chickens were introduced and were allowed to co-habitate with infected ducks. Tissues were collected from euthanized or acutely dead birds at 5 dpi/dpc, and 5 µm tissue sections were treated for the detection of virus antigen as described in detail in Materials and Methods. Insets show higher magnification of affected areas.

**Fig 9:**
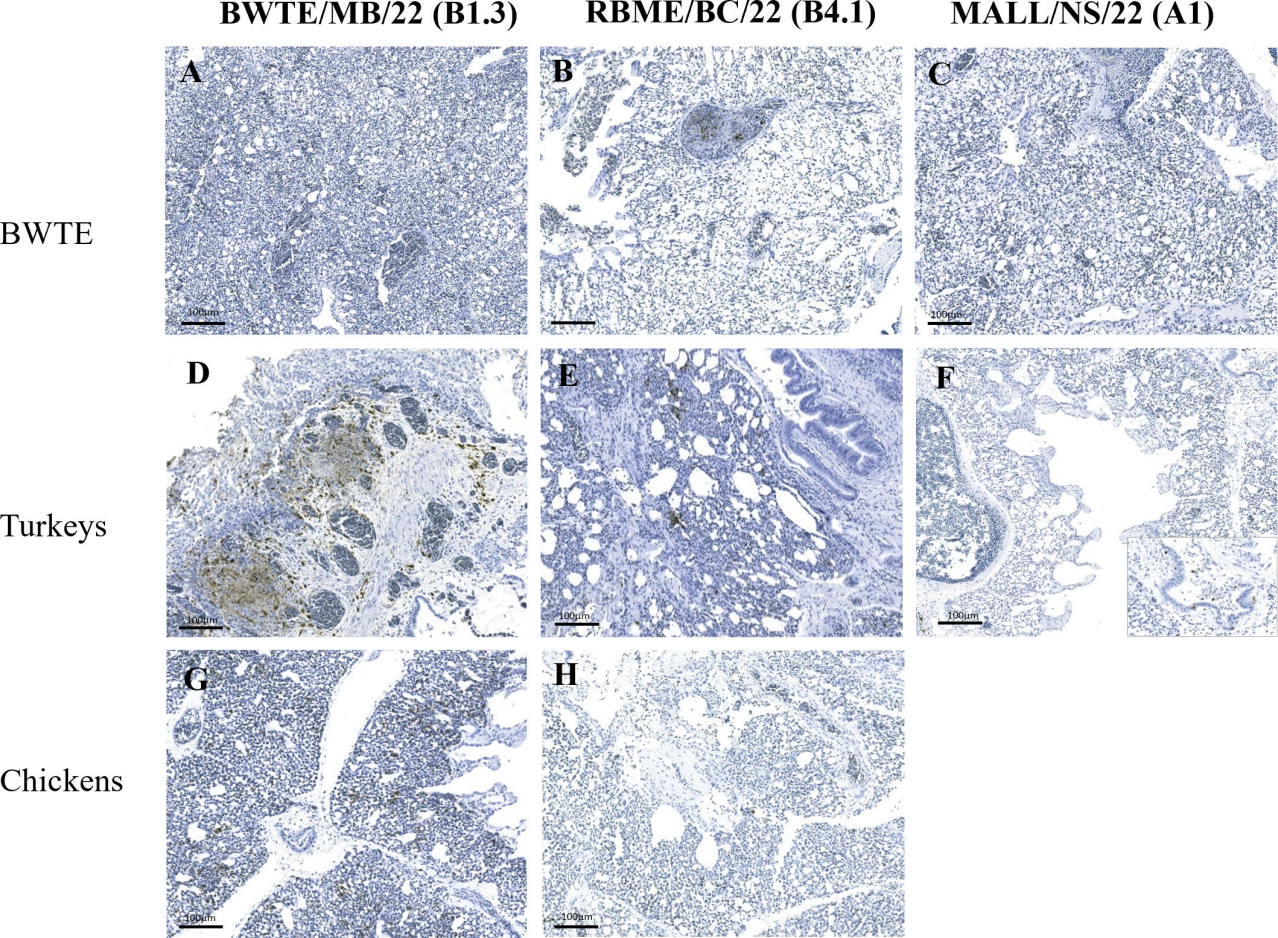
Detection of influenza A virus antigen in lung tissues of infected BWTE (**A through C**), contact turkeys (**D through F**), and contact SPF chickens (**G, H**) with A(H5N1) clade 2.3.4.4b HPAI viruses isolated in 2022. BWTE were challenged with 1 × 10^6^ TCID_50_ of A(H5N1) HPAI viruses by the intranasal route. Twenty-four hours post-infection, turkeys and SPF chickens were introduced and were allowed to cohabitate with infected ducks. Tissues were collected from euthanized or acutely dead birds at 5 dpi/dpc, and 5 µm tissue sections were treated for the detection of virus antigen as described in detail in Materials and Methods. In F, the inset shows the area magnified.

Immunostaining indicated influenza viral antigen-laden neurons in brain sections of BWTE infected with the reassortant viruses (Fig. 8A and B). Virus antigens were undetected in the brain sections from BWTE infected with MALL/NS/22 (Fig. 8C). Although the patterns of virus antigen distribution varied in the brain, the three viruses were also found neuroinvasive in both turkeys (Fig. 8D through F) and chickens (Fig. 8G and H). Virus antigens were scant in the lungs of BWTE infected with BWTE/MB/22 (Fig. 9A); however, staining was moderate to extensive in the lungs of contact turkeys (Fig. 9D) and chickens (Fig. 9G). Scant to mild immunostaining was observed in the lungs of BWTE, turkeys, and chickens with RBME/BC/22 infection (Fig. 9B, E, and H). Weakly-stained cells were detected in the lungs of BWTE and turkeys infected with MALL/NS/22 (Fig. 9C and F).

Mild to moderate histological lesions in heart and liver tissues and weak to abundant detection of influenza antigen were observed in BWTE and contact chickens and turkeys infected with reassortant viruses (Table S2). No lesions or viral antigen were detected in animals infected with the fully Eurasian A(H5N1) virus.

### Seroconversion in survivor birds

Serum samples collected from BWTE had detectable antibodies in HI and NP-cELISA tests, and the HI titers in all groups ranged from 128 to 512. None of the surviving contact chickens had detectable HI or NP antibodies, demonstrating the absence of efficient contact transmission.

### Inter- and intra-host evolution

To explore within-host viral diversity, we assessed mutations during transmission by performing next-generation sequencing on viruses from infected BWTE (3 dpi) and contact turkeys and chickens (6–8 dpc). While all viruses showed some level of nucleotide diversity, there was no association between nucleotide mutations and dpi/dpc or host species (Fig. 1) (Fig. S1). The accumulated nucleotide mutations in the 3 dpi and 6–8 dpc samples did not result in any gallinaceous-adaptive amino acid substitutions (i.e., no deletion of a certain amino acid string in the neuraminidase stalk region). Thus, A(H5N1)-infected ducks transmitting to either turkeys or chickens did not exhibit host-switching and subsequent virus transmission.

## DISCUSSION

The pervasive and recurrent emergence of A(H5N1) viruses within the Americas has resulted in the widespread distribution of A(H5N1) clade 2.3.4.4b viruses, carrying internal genes derived from North American lineage LPAI viruses while retaining conserved surface proteins from Eurasian strains (3). These viruses constitute a significant proportion of the A(H5N1) virus population associated with HPAI outbreaks in domestic poultry, wild aquatic birds, and mammalian species (3, 37). In the current study, three distinct genotype A(H5N1) clade 2.3.4.4b viruses were isolated, and their phylogenetic characteristics and host switching properties were determined. Their pathogenicity was then assessed in BWTE along with the capacity for viral transmission to gallinaceous poultry. Compared to most experiments, the dabbling ducks used in the current study were relatively older and are expected to play crucial roles in HPAI virus dispersion. The pathogenicity of GsGd lineage A(H5N1) viruses in birds is influenced by factors, including the age (38), species (30, 39, 40), and immune status of the avian hosts (41), and the virus subtypes and clades (42, 43). The BWTE selected for this experimental study were of similar age, collected from the same aggregation sites shortly after fledging. These birds were housed in high-containment animal cubicles for 8 weeks before the challenge. Given the ubiquitous nature of IAV in these reservoir species, the BWTE may have possessed maternal antibodies that waned during the 8 week adaptation period. Consequently, these IAV-antibody-free BWTE exhibited significant variations in clinical manifestations and pathological outcomes when challenged with different genotypes of A(H5N1) clade 2.3.4.4b viruses.

Within the BWTE model, these viruses induced a range of infections from lethal to subclinical infections. Notably, the reassortant clade 2.3.4.4b virus designated BWTE/MB/22 (B1.3 genotype) demonstrated marked pathogenicity in BWTE aged over 12 weeks. Although pathogenicity trait assessments in Anseriformes, such as ducks, have been primarily conducted on juvenile groups (11, 26, 42), the role of juvenile birds in the epidemiology of HPAI remains inadequately defined. In juvenile wild ducks, neurotropic A(H5N1) clade 2.2 viruses, which were isolated in 2005, as well as A(H5Nx) clade 2.3.4.4b viruses, which have been circulating since 2020, have been demonstrated to cause a rapid onset of disease progression leading to fatal outcomes (11, 26, 27, 38, 39). This is in stark contrast to the less severe and often asymptomatic manifestations noted in mature or adult mallards when experimentally infected with either clade 2.2 or A(H5N6) clade 2.3.4.4b viruses (11). The pathogenic phenotype traits observed, which included neurological and hepatotropic complications, ultimately resulted in mortality or euthanasia in 66.7% of BWTE infected with the BWTE/MB/22 strain (B1.3 genotype). This result revealed that GsGd lineage A(H5Nx) viruses appear to have acquired increased virulence throughout their emergence in various host species (39, 44). This figure may represent the highest mortality rate documented for relatively older ducks when compared to similar data from infection studies involving historical GsGd lineage A(H5Nx) viruses (10, 11). This pathogenicity is reflected in our host dynamics analyses, as host transitions for BWTE/MB/22 (B1.3 genotype) were almost exclusively from Anseriformes to carnivores and scavengers, resulting in fatal outcomes. The pathogenic consequences associated with neuro- and hepato-tropic A(H5Nx) clades 2.3.4.4b and 2.3.4.4c infections in juvenile Muscovy and Pekin ducks reiterated the findings obtained from juvenile wild ducks, characterized by substantial viral loads and overt clinical manifestations that culminated in lethal outcomes (40).

In our present study, the reassortant virus RBME/BC/22 (B4.1 genotype) was found to induce 12.5% mortality in BWTE, accompanied by clear indicators of systemic viral spread. However, this infection resulted in fewer multifocal necrotic and inflammatory lesions in the brain and other visceral organs compared to those observed in infections with BWTE/MB/22 (B1.3 genotype). These findings collectively support the hypothesis that young adult BWTE may demonstrate a degree of resistance to infections still caused by some A(H5Nx) HPAI viruses. The wholly Eurasian MALL/NS/22 (A1 genotype) virus caused subclinical or silent infections. This highly waterfowl-adapted virus replicated locally within the respiratory and gastrointestinal tracts without inducing clinical signs or mortality. This nature probably provides MALL/NS/22 (A1 genotype) virus with significant virtues for persistence and adaptation in wild birds. Conversely, a wholly Eurasian A(H5N1) clade 2.3.4.4b virus isolated during the 2021/2022 period (initial wave of incursion) in the United States was associated with symptomatic disease and 40% mortality in experimental juvenile mallards (45). Notably, 60% of the ducks remained asymptomatic despite shedding considerable quantities of the virus (45). Extrapolating pathogenicity data from juvenile mallards may not accurately represent the responses in BWTE, but it is reasonable to expect that the wholly Eurasian A(H5N1) strain, MALL/NS/22, could have induced severe pathological outcomes and mortality if 2-week-old juvenile BWTE were infected. Additionally, other clades, including the highly wild bird-adapted A(H5Nx) clade 2.3.4.4a viruses, isolated between 2014 and 2015, have been documented to cause less severe to non-lethal infections in both wild juvenile and mature ducks (26, 27) and in domesticated ducks (30, 40).

This experimental study using the BWTE/MB/22 (B1.3 genotype) virus provides substantial evidence that reassortant viruses containing an increased number of North American gene segments exhibited enhanced virulence in BWTE. Of the three viruses evaluated in this study, BWTE/MB/22 (B1.3 genotype) sequences are the least commonly detected from wild-bird populations (GISAID database). However, our phylodynamic analyses find disproportionately high transmission rates of BWTE/MB/22 (harboring 4 EA segments and 4 NAm segments) to mammals, raptors, and corvids, relative to the other two viruses evaluated. Using reverse genetic systems for A(H5Nx) viruses, previous studies showed that modifications that involved a minimal number of gene segment combinations altered key pathogenicity markers, including systemic virus spread, clinical presentations, mortality, and transmission of the recombinant viruses. Notably, a reverse genetically derived (RG) H5N8 virus constructed from a 2016 A(H5N8) clade 2.3.4.4b backbone with insertions of PB2, NP, and M genes derived from a 2014 A(H5N8) clade 2.3.4.4a virus had markedly reduced pathogenicity in mallards. This was attributed to severely impacted polymerase activity (46). Furthermore, the infectivity of an RG 2005 A(H5N1) virus generated from a GsGd A(H5N1) genetic backbone comprising PB2, PA, HA, NP, and NS genes from another virus investigated in Pekin ducks elucidated alterations in pathogenic phenotype. The resulting virus caused higher mortality compared to the parental A(H5N1) virus in Pekin ducks (47). Since the present pathogenesis study was limited to naturally occurring reassortant viruses, the molecular mechanisms of increased pathogenicity of A(H5N1) clade 2.3.4.4b virus could be elucidated in further investigation using RG viruses generated from recent isolates by stepwise substitutions of the internal genes between Eurasian and North American viruses. It is noteworthy that the inheritance of a complete polymerase complex from the LPAI viruses, already optimized for fitness to replicate in ducks, may contribute to the enhanced host switching and possibly pathogenicity observed in some of the viruses used in our study (37).

The differential pathogenicity of the two reassortant viruses (B1.3 and B4.1 genotypes) was highlighted by higher virus shedding in the OP and CL swabs, along with a prolonged shedding duration. This dynamic of virus shedding was consistent across dabbling and diving ducks infected with earlier A(H5Nx) clade 2.3.4.4 and 2.3.2.1 viruses (11, 27, 42) and recent A(H5N1) clade 2.3.4.4b virus (45). In our direct transmission study, efficient transmission to contact turkeys led to a significant deterioration in the health conditions, resulting in 100% mortality of turkeys across all three viruses. All exposed contact turkeys shed a significant amount of virus via the OP and CL routes. This outcome is not unexpected, given that turkeys are among the most susceptible species to HPAI viruses within the Galliformes order (48). In a co-housing study, contact transmission is highly likely to have occurred through the fecal-oral route and aerosol droplets. The higher quantities of virus excreted by the BWTE have the potential to contaminate their surrounding environment. Measurement of virus levels in environmental specimens (fecal droppings and water) and air samples was not performed in this study, but typically, low levels to undetectable viral RNA were described in environmental samples in a recent study evaluating the transmission dynamics of A(H5N1) clade 2.3.4.4b virus in mallards and chickens (33). In contrast, chickens co-mingled with BWTE infected with BWTE/MB/22 (B1.3 genotype) and RBME/BC/22 (B4.1 genotype) experienced overall mortality of 60% and 33.3%, respectively. Post-contact oral and cloacal swabs revealed considerably higher quantities of viruses. The hallmarks of infection in naïve turkeys and chickens in the reassortant viruses were viral shedding followed by mortality of all shedders due to conspicuous tissue pathologies and influenza antigen spread in systemic organs. Consistently, infections of HPAI viruses in gallinaceous poultry have resulted in high virus load found in both OP and CL swabs (49). An increasing number of studies have documented similar transmission dynamics occurring in turkeys that were co-mingled with GsGd HPAI virus-infected Pekin ducks (30, 50).

No evidence was found regarding the transmission of MALL/NS/22 (A1 genotype) to chickens following co-mingling with infected BWTE, despite the high levels of virus shedding by the infected ducks. All contact chickens remained healthy until the conclusion of the experiments, and the diagnostic quantitative PCR (qPCR) assays did not detect viral RNA in the OP and CL swabs throughout the study period. Serum samples analyzed for nucleoprotein and hemagglutination-inhibiting antibodies presented negative results, confirming they were never infected by the virus. With its fully Eurasian gene, MALL/NS/22 may have retained its waterfowl adaptation compared to the reassortant viruses, hence restricting transmissibility to chickens at least in experimental studies. Challenge studies have demonstrated an impaired or complete loss of transmission potential to contact gallinaceous poultry of contemporary A(H5Nx) clade 2.3.4.4b and clade 2.3.4.4c HPAI viruses isolated from wild ducks, including under higher challenge dose experiments (27, 33, 51). In contrast, a Pekin duck challenge experiment involving the A(H5N8) clade 2.3.4.4a virus documented a direct contact transmission to chickens within a co-habitation context, suggesting that the origin of the specific virus subtypes and the species of infected donors could significantly influence transmission efficacy. Furthermore, onward transmission from infected contact chickens to newly introduced naïve chickens did not occur upon the removal of directly infected Pekin ducks from the environment, indicating a lack of host-switching (30). Notably, as the transmission and viral shedding in chickens were delayed in our study by several days, a potential host switching was expected; however, this was not the case. No evidence of Galliformes-associated genetic alterations (stalk deletions in the NA gene) or significant single-nucleotide polymorphisms was noted during the transmission process, as the isolates from each species maintained their original gene sequences. The likely direction of virus transmission was from infected BWTE to chickens or turkeys, without host switching among chickens or turkeys. A prerequisite for efficient and dynamic transmission of HPAI viruses to poultry, specifically chickens, requires virus adaptation into terrestrial gallinaceous poultry. The A(H5N2) clade 2.3.4.4c HPAI virus, isolated in the United States during the 2014/2015 outbreak, exemplifies such adaptation, being readily transmissible among chicken flocks (31, 51). Various factors at the farm level could modify HPAI infection outcomes, including rapid farm-to-farm spread and higher lethality traits of A(H5N1) clade 2.3.4.4b viruses that may constrain adaptation of currently circulating A(H5N1) HPAI viruses in chickens.

Our results show that BWTE can be used to explore the pathogenicity of A(H5N1) viruses and their roles as dispersion vectors for HPAI viruses. Compared to the original Eurasian A(H5N1) virus, reassortants containing NAm lineage gene segments had an enhanced virulence phenotype in BWTE and readily transmitted to naïve contact domestic poultry. Detailed immunologic profiling in dabbling ducks of infections with clade 2.3.4.4b A(H5N1) could provide evidence of differences in pathogenicity between various emerging genotypes of HPAI viruses (52).

## MATERIALS AND METHODS

### Virus isolation

Three A(H5N1) clade 2.3.4.4b viruses circulating in Canada during the 2022 epidemic (9) were isolated from OP and CL swabs in embryonated SPF chicken eggs. The three viruses represented the most common genotypes circulating in 2022 (A1, B1.3, and B4.1 genotypes), isolated from different dabbling duck species. The three viruses were isolated in the first passage in eggs, and the allantoic fluid was harvested on day 2 post-inoculation. The A/BWTE/MB/FAV913/2022 (BWTE/MB/22) belonged to B1.3 genotype viruses and was an isolate from a deceased blue-winged teal (*Anas discors;* BWTE) from Manitoba (9). The A/RBME/BC/FAV899-13/2022 (RBME/BC/22) was an isolate from a red-breasted merganser (*Mergus serrator*; RBME) from British Columbia and genotyped as B4.1 virus. The A/MALL/NS/FAV102-3/2022 (MALL/NS/22) was isolated from mallard duck (*Anas platyrhynchos; MALL*) from Nova Scotia and genotyped as A1 virus. Genomic procedures related to IAV sequencing were described in our article (53). The three viruses were genotyped using GenoFLU software developed by the USDA (https://github.com/USDA-VS/GenoFLU). Full genome sequences are deposited in the Global Initiative on Sharing All Influenza Data (GISAID).

### Experimental design

BWTE were housed in containment level three plus (CL3-Ag) animal cubicles at NCFAD, Winnipeg, for 8 weeks. Feed and water were *ad libitum,* their feed was formulated to include green grasses, and they were provided with wading pools. BWTE were determined free from active IAV shedding or antibodies prior to the experiment by the methods described previously (12, 54). At about 12 weeks of age, they were divided into three groups (*n* = 9–10 BWTE/group), and each group was intranasally infected with 10^6^ TCID_50_ of A(H5N1) clade 2.3.4.4b virus described above in 250 uL Dulbecco’s modified Eagle medium (125 uL per nostril). Twenty-four hours post-infection, 4-week-old domestic turkeys (*n* = 5–6) and SPF chickens (*n* = 5–6, CFIA, Ottawa, Canada) were introduced as contacts to each infected BWTE group to assess transmission. The three species were co-housed and roamed freely in the cubicles. This experimental approach mimics intimate domestic poultry-wild bird interfaces as experienced in the first Canadian A(H5N1) outbreak in 2021 in a multi-species exhibition farm, facilitating the transmission of highly duck-adapted viruses (1). Birds were monitored daily for clinical signs and mortality and euthanized when reaching the humane endpoint. OP and CL swabs were collected in 2 mL universal virus transport medium (Copan, CA, USA) on 3, 5, 7, 9, and 14 dpi from BWTE and on 2, 4, 6, 8, and 13 dpc from chickens and turkeys to assess virus shedding. Sets of brain, lung, liver, and heart tissues were collected from BWTE sacrificed at 3 and 5 dpi and from euthanized or dead turkeys/chickens for histology and IHC. Serum samples were collected from surviving birds at the end of the experiment (21 dpi/20 dpc).

### Quantification of virus shedding

A standard curve was generated from a 10-fold dilution series of an *in vitro*-synthesized RNA template from the matrix gene of IAV to calculate matrix gene-specific RNA copy numbers in the samples (OP and CL swabs) based on the linear regression of the standard curve in the LightCycler480 System (Roche Diagnostics). The results were expressed as log_10_ viral RNA copy numbers per reaction.

### Histopathology and virus antigen detection

Sets of brain, lung, liver, and heart tissue samples were collected from BWTE, turkeys, and chickens and fixed in 10% neutral phosphate-buffered formalin. The samples were trimmed, embedded in paraffin, sectioned at 5 µm, and stained with Hematoxylin and eosin to evaluate microscopic lesions. IHC was used to localize IAV antigen in tissues as described (12, 54). One BWTE was examined per timed euthanasia (3 and 5 dpi), and a few BWTE that died or were euthanized at humane endpoints. The extent of antigen loads in IHC-stained tissue sections (shown in brown coloration) was expressed as described in Löndt et al. (24) with modification: −, no virus antigen; +, less than 25 cells stained; ++, moderate number of scattered cells stained for the virus (>25%–50%); and +++, often widespread and abundantly stained cells (>50%) per microscopic field (10×).

### Antibody detection

The IAV antibody responses in surviving birds (BWTE and chickens) were determined with the nucleoprotein (NP) specific c-ELISA as described (54). To determine anti-H5 hemagglutinin antibodies, A(H5N1) clade 2.3.4.4b virus (A/Turkey/ON/FAV162-144/2022) was used as a reference virus. Hemagglutination inhibition test was performed as described (55), and HI titers were determined as the reciprocal of the highest serum dilution resulting in complete inhibition of 4 HAU of the virus.

### Genome sequencing and single-nucleotide polymorphism analysis

Viral sequences from this study were combined with previously published sequences of the same genotype (37). For each genotype, whole-genome sequences were aligned using MAFFT v7.49 and used to estimate a time-scaled phylogenetic tree using BEAST v1.10.4 with a GMRF Bayesian Skyride tree prior, the best fitting nucleotide substitution model as determined by ModelFinder, and a relaxed uncorrelated molecular clock rate with lognormal mean distribution. Hosts were binned into five groups: Anseriformes, Charadriformes, domestic birds, carnivores, and scavengers (mesocarnivorous mammals, raptors, and corvids), and others (Suliformes, Podicipediformes, and Pelecaniformes). Host transitions were estimated by adding the host group of each tree tip as a discrete character trait. Host transitions were reconstructed for all nodes of the tree by the asymmetric substitution model, and social networks inferred BSSVS. BEAST xml files were edited to log Markov rewards and the complete Markov jump history for each host group. Two independent Markov chain Monte Carlo chains (200,000,000 steps, sampled every 20,000) were run for each genotype. Each chain was assessed for convergence based on equivalent sampling size >200 using Tracer v1.7.279, and the first 10%–50% of samples from each chain were discarded as burn-in, depending on the time to convergence. Post-burn-in samples for each segment were combined using LogCombiner v1.10.4, and maximum clade credibility trees were produced using TreeAnnotator v1.10.4. The posterior distribution of indicator values from the BSSVS procedure was used to conduct Bayes factor (BF) tests to quantify statistical support for host transitions using SpreaD3. Host transitions with BF <3.0 were excluded from the data set. The genetic polymorphisms during experimental infection of BWTE and transmission to domestic poultry were assessed by comparing virus sequences obtained from OP swabs of BWTE at 3 dpi and from contact chickens and turkeys at 6–8 dpc. Sequences were compared to the original challenge viruses. Genomic procedures related to sequencing were described in a previous section.

### Statistics

Statistical analysis was conducted on GraphPad Prism (Version 7, GraphPad, La Jolla, CA). Kruskal–Wallis one-way non-parametric analysis of variance was used to analyze viral shedding in OP and CL swabs. Comparisons were made between viruses at specific time points. Survival curves were computed using a log-rank (Mantel-Cox) test. Data were given as the mean ± SEM. *P* < 0.05 indicates a significant difference in virus shedding from infected birds.

## Data Availability

Full genome sequences are deposited under accession numbers EPI_ISL_17394090, EPI_ISL_19154026, and EPI_ISL_19145108 (https://doi.org/10.55876/gis8.250512ov) in the Global Initiative on Sharing All Influenza Data (GISAID). All data are presented in the paper and available in the supplemental files.

## SUPPLEMENTAL MATERIAL

Supplemental Material msphere.00021-25-s0001.pdf Tables S1 and S2; Figure S1.

ASM does not own the copyrights to Supplemental Material that may be linked to, or accessed through, an article. The authors have granted ASM a non-exclusive, world-wide license to publish the Supplemental Material files. Please contact the corresponding author directly for reuse.
