# Supplementary material for: Comparative pathogenicity of three A(H5N1) clade 2.3.4.4b HPAI viruses in blue-winged teal and transmission to domestic poultry
Source: mSphere. 2025 May 22;10(6):e00021-25. doi: 10.1128/msphere.00021-25 (PMC12188715; doi:10.1128/msphere.00021-25)
Supplement: Supplemental Material — Tables S1 and S2; Figure S1. [file msphere.00021-25-s0001.pdf]

TABLE S1. Pathological alteration and immunostaining for influenza viral antigen in tissues of BWTE infected with A(H5N1) clade 2.3.4.4b HPAI viruses.

Parameters	Tissues	3-days post-infection		
		**BWTE/MB/22 (B1.3 genotype)**	**RBME/BC/22 (B4.1 genotype)**	**MALL/NS/22 (A1 genotype)**
Pathology	Brain	Multifocal areas of gliosis associated with moderate numbers of necrotic neurons	Occasional perivascular lymphocytic infiltration	Mild ↑glial cells, occasional necrotic neurons
	Lung	Mild perivascular cuffing, mild inflammatory cell infiltrations in peribronchiolar areas	Congestion	Peri-bronchial mild pneumonia
	Heart	No lesion	No lesion	No lesion
IHC	Brain	+++	-	+
	Lung	+	_	+
	Heart	+	-	-

TABLE S2. Pathological alteration and immunostaining for influenza viral antigen in tissues of BWTE and poultry infected with A(H5N1) clade 2.3.4.4b HPAI viruses.

		**5/7-Days post-infection**								
**Parameters**	**Tissues**	**BWTE/MB/22 (B1.3 genotype)**			**RBME/BC/22 (B4.1 genotype)**			**MALL/NS/22 (A1 genotype)**		
Pathology		**BWTE**	**Turkey**	**Chicken**	**BWTE**	**Turkey**	**Chicken**	**BWTE**	**Turkey**	**Chicken**
	Heart	No lesion	No lesion	Mild inflammation	No lesion	Mild multifocal myocarditis	Mild focal myocarditis	No lesion	No lesion	NA
	Liver	Moderate, multifocal necrosis and inflammation	No lesion	Mild multifocal hepatitis	Mild multifocal hepatitis	Mild multifocal hepatitis	No lesion	No lesion	No lesion	NA
IHC	Heart	-	++	+++	-	++	-	-	-	NA
	Liver	++	+++	++	++	+	-	-	-	NA

Histologic descriptions for contact chickens in the MALL/NS/22 group were not provided as none of them died or were euthanized.

IHC = Immunohistochemistry

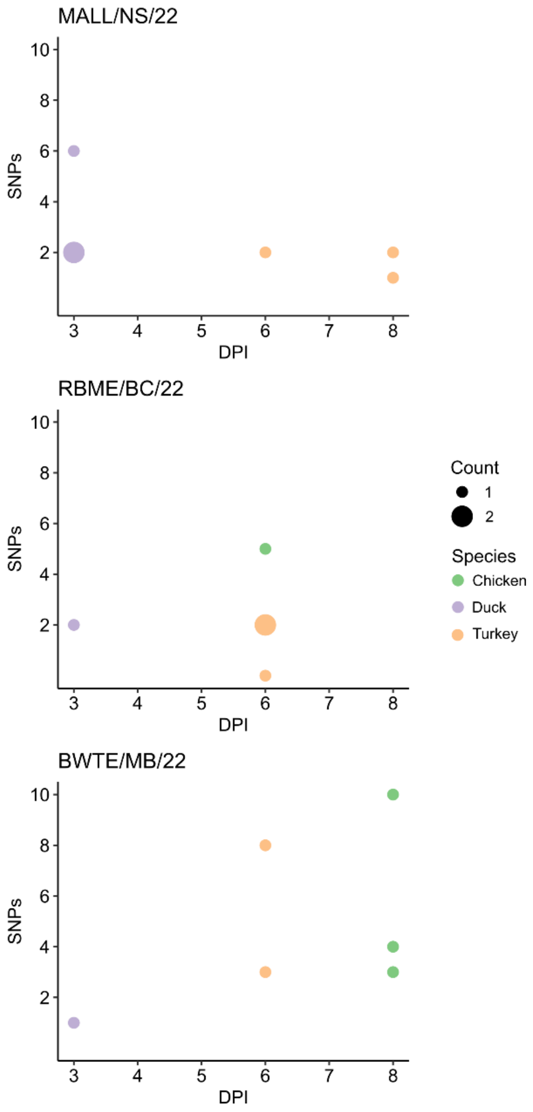

FIG S1. Genetic polymorphism assessment of A(H5N1) viruses in infected ducks/turkeys/chickens during the transmission events. Oropharyngeal swabs from infected ducks at 3 dpi and from contact chickens and turkeys at 6-8 dpc were compared to the reference virus sequences used for the challenge to analyze SNPs.
